# Apotropaic Practices and the Undead: A Biogeochemical Assessment of Deviant Burials in Post-Medieval Poland

**DOI:** 10.1371/journal.pone.0113564

**Published:** 2014-11-26

**Authors:** Lesley A. Gregoricka, Tracy K. Betsinger, Amy B. Scott, Marek Polcyn

**Affiliations:** 1 Department of Sociology, Anthropology, & Social Work, University of South Alabama, Mobile, Alabama, United States of America; 2 Department of Anthropology, SUNY College at Oneonta, Oneonta, New York, United States of America; 3 Department of Anthropology, University of Manitoba, Winnipeg, Manitoba, Canada; 4 Department of Anthropology, Lakehead University, Thunder Bay, Ontario, Canada; University of South Carolina, United States of America

## Abstract

Apotropaic observances-traditional practices intended to prevent evil-were not uncommon in post-medieval Poland, and included specific treatment of the dead for those considered at risk for becoming vampires. Excavations at the Drawsko 1 cemetery (17^th^–18^th^ c. AD) have revealed multiple examples (n = 6) of such deviant burials amidst hundreds of normative interments. While historic records describe the many potential reasons why some were more susceptible to vampirism than others, no study has attempted to discern differences in social identity between individuals within standard and deviant burials using biogeochemical analyses of human skeletal remains. The hypothesis that the individuals selected for apotropaic burial rites were non-local immigrants whose geographic origins differed from the local community was tested using radiogenic strontium isotope ratios from archaeological dental enamel. ^87^Sr/^86^Sr ratios ( = 0.7112±0.0006, 1σ) from the permanent molars of 60 individuals reflect a predominantly local population, with all individuals interred as potential vampires exhibiting local strontium isotope ratios. These data indicate that those targeted for apotropaic practices were not migrants to the region, but instead, represented local individuals whose social identity or manner of death marked them with suspicion in some other way. Cholera epidemics that swept across much of Eastern Europe during the 17^th^ century may provide one alternate explanation as to the reason behind these apotropaic mortuary customs, as the first person to die from an infectious disease outbreak was presumed more likely to return from the dead as a vampire.

## Introduction

Deviant burials in the archaeological record refer specifically to those interments that differ from standard burial rites for a given community or culture [1]. While normative mortuary rituals offer valuable insight into social constructs of individual and group self among the living [2], deviant burials provide information on identity from a very different perspective – one in which normal burial was denied due to some social condition during life or as a result of a specific kind of death [3]. As treatment of the dead can reflect how the living view (or more idealistically, want to view) themselves, a study of deviant burial practices conversely illustrates action taken against the deceased because of a social identity (endowed during life or after death) incongruous with that of the larger culture. Such action may include the use of apotropaics – practices undertaken or objects utilized to prevent evil – within the grave itself [4]. These apotropaic rites play an important role in the interment of individuals suspected of turning into vampires throughout Eastern Europe in the medieval and post-medieval periods [4],[5],[6].

In addition to apotropaics, the skeletons of those receiving non-normative mortuary treatment are an especially rich source of data because the body acts not just as a biological representation of personhood, but as an entity shaped by social processes and affected by culture [7],[8],[9]. In particular, biogeochemical analyses of skeletal tissues reflect the impact of the social on the biological – most notably, the incorporation of stable isotopes into dental enamel and bone, which help archaeologists to elucidate aspects of social life in the past, including patterns of mobility and geographic origins. Subsequently, strontium isotope analysis was undertaken at the post-medieval cemetery site of Drawsko 1 (17^th^–18^th^ c. AD) in northwestern Poland to better discern social representations of identity as reflected in the differential burial of those designated as potential vampires in death by apotropaics. While individuals targeted as vampires might have been selected for a variety of reasons, the sociopolitical events of the post-medieval period in Poland, including well-documented, large-scale immigration to the region during this time [e.g., 10, 11, 12], suggested that those interred with anti-vampiristic apotropaics might be non-local migrants whose outsider status marked them with the potential to return from the dead. Correspondingly, it was hypothesized that the dental enamel of the six individuals buried as possible vampires would exhibit non-local strontium signatures relative to those contained within ‘normal’ graves, indicative of immigration to the region later in life.

## The Vampire in Eastern Europe

The notion of vampires, revenants, or the undead has a long history in both cultural folklore and literary works as well as in contemporary popular media. In the Slavic region of Eastern Europe, the vampire, or reanimated corpse, was established as early as the 11^th^ century AD [13],[14]. The term “vampire” probably originated from the Slavic expression for revenants, including *vampir* and *upir/upyr/upiór*, also similar to *uber*, the Turkish word for witches [15]. The vampire myth, however, likely originated much earlier and is a known mythic entity among the ancient Romans, Egyptians, and Greeks [15]. While the term “vampire” has been used to refer to a variety of comparable mythological beings from diverse cultures [4],[15], it is used here to refer to the beings of early Polish folklore: an unclean spirit that reanimates a corpse and wreaks havoc on the living [16],[17]. Here, we use “vampire” to refer to those who were *at risk* of becoming reanimated.

In Polish folklore, the soul and the body are distinct entities that separate upon a person’s death. Souls, the majority of which are harmless, leave the body and continue to inhabit the earth for 40 days after death [13]. However, a small minority of these souls were seen as a direct threat to the living and at risk of becoming a vampire, particularly those who were marginalized in life for having an unusual physical appearance, practicing witchcraft, perishing first during an epidemic, committing suicide, being unbaptized or born out of wedlock, or being an outsider to the community [4],[5],[13],[16],[18],[19].

In order to prevent the soul of the deceased from reanimating a corpse as a vampire, specific burial rites were undertaken [20]. These burial customs are identifiable in the archaeological record, enabling researchers to distinguish vampire burials from the general populous. The inclusion of apotropaics, or grave goods that prevent evil or barricade an individual within the grave, is one of the most common anti-vampiristic methods employed [4],[5],[6]. For example, the inclusion of sharp instruments such as sickles or scythes placed over the neck and abdomen was believed to destroy the physical body if a vampire attempted to rise from the grave [4],[6].

While vampire folklore originated as part of pagan religious traditions across Eastern Europe, the adoption of Roman Catholicism in the 10^th^ century did not curb belief in vampires throughout the medieval and post-medieval periods [10],[21]. To promote the acceptance of Catholicism, early medieval Christianity may have syncretistically incorporated pagan beliefs – including ancestor worship and a belief in vampirism – although there is much debate regarding the degree of assimilation of these pagan traditions [10],[22],[23]. Nevertheless, support for religious diversity was apparent as late as the 16^th^ century, when the Warsaw Confederation document of AD 1573 clearly outlined religious tolerance and established religious freedom in Poland [24],[25],[26]. However, such tolerance seemed to ebb during the Catholic Reform of the 17^th^ and 18^th^ centuries, when Parliamentarian acts were passed that prevented non-Catholics from achieving certain government positions and prohibited the renunciation of Catholicism [25].

The turbulent nature of the Reformation period in Poland may have enabled pagan beliefs regarding vampirism and the undead to persist in what had become a predominantly Christian social order [10]. In what seems to be in direct conflict to the Reformation, some priests apparently accepted the inclusion of pagan mortuary customs in conjunction with Christian funeral rituals as long as Christian rites were maintained [5]. In particular, pagan burial traditions related to fear of revenants and the reanimation of the dead persisted well into the post-medieval period [18],[20]. This acceptance of pagan burial rites may have actually benefited the Church in terms of encouraging adherence to Church doctrine [27].

## Apotropaic Practices at Drawsko

Drawsko is a rural settlement site in northwestern Poland that has been continuously occupied since the medieval period [28],[29]. Situated on the banks of the Noteć River, Drawsko has an associated post-medieval cemetery site located outside of the contemporary village on its eastern edge, designated Drawsko 1 [28],[29]. The Drawsko 1 cemetery has been dated to the 17^th^ and 18^th^ centuries AD based predominantly on coins recovered from the site. Initial excavations of the cemetery began in 1929; however, systematic excavations did not occur until 2008 [28],[29]. The cemetery is particularly unusual for this time period because of its far-removed location from Drawsko itself [27].

**Figure 1:**
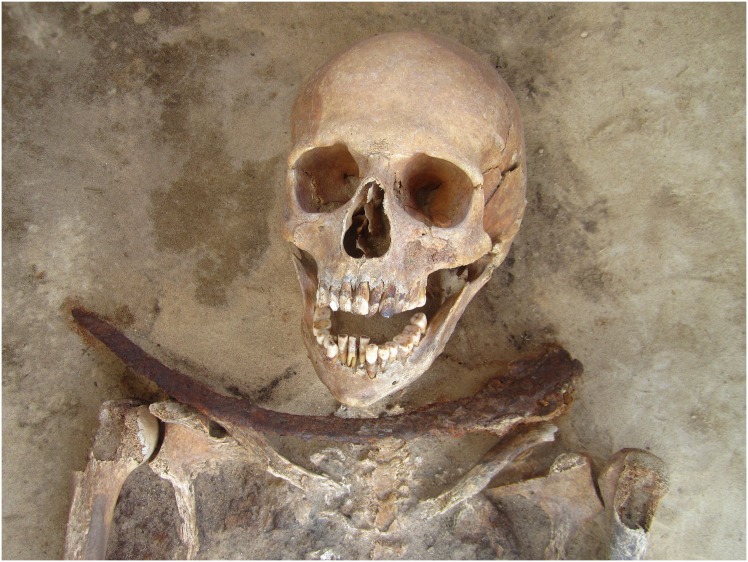
Individual 49/2012 (30–39 year old female) with a sickle placed across the neck.

**Figure 2:**
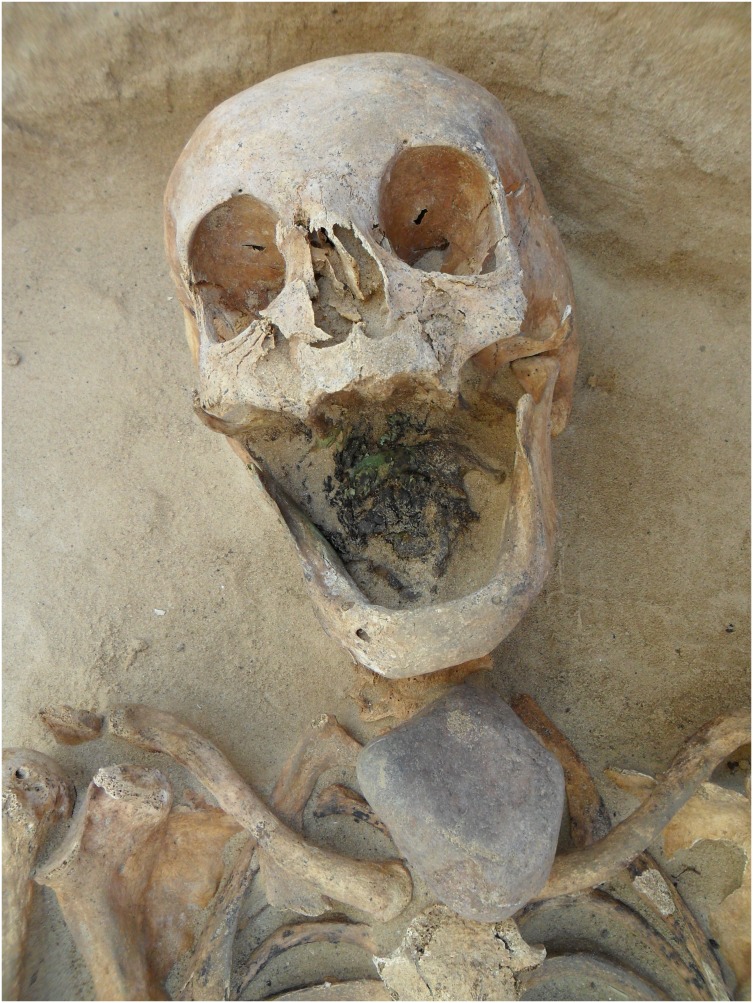
Individual 60/2010 (45–49 year old female) with a stone placed directly on top of the throat.

Approximately 285 human skeletons were recovered from Drawsko 1 between 2008–2012, representing individuals of all age categories and of both sexes. The remains are generally well preserved, and burials consist of individual interments, frequently lain in wooden coffins. All burials are primary inhumations with no evidence of secondary disturbances. Six of these interments have been classified as deviant (vampire) burials due to their non-normative mortuary features and apotropaic inclusions involving anti-vampiristic alterations. These include one adult male (28/2008), one late adolescent female (6/2012), three adult females (24/2009; 60/2010; 49/2012), and a subadult of unknown sex (29/2008); such demographic diversity indicates that apotropaic mortuary practices against vampirism were not sex or age-specific. Of these six individuals, five were interred with a sickle placed across the throat or abdomen, intended to remove the head or open the gut should they attempt to rise from the grave (**Figure 1**). Two individuals also had large stones positioned beneath their chins, likely as a preventative measure to keep the individual from biting others [4] or to block the throat so that the individual was unable feed on the living [30] (**Figure 2**). Interestingly, these burials are not segregated in the cemetery but were placed amidst non-deviant interments.

Other grave goods at Drawsko included medals and coins. Three medals, two of which depict St. Benedict, were found associated with three non-deviant burials. The image of St. Benedict and the cross represents a mortuary inclusion thought to ward off evil spirits, and was perhaps intended as an anti-vampiristic measure [31]. A third medal had no visible markings. Coins (n = 119, represented in 36% of excavated graves) also signify an important apotropaic placed with the dead, and were thought to protect the body from evil spirits [4]. While these charms were sometimes simply laid on or near the body, many of these coins were placed under the tongue, not only to prevent a malicious spirit from entering the body through the mouth, but also to give the undead something to bite to dissuade them from feeding on the living [4],[5],[32].

Previous bioarchaeological research examining the health of the individuals recovered from the cemetery at Drawsko 1 has yielded no statistically significant patterns of disease, stress, or trauma between deviant and non-deviant burial groups [33]. These findings suggest that biological factors were not prime determinants in an individual’s risk of becoming a vampire, at least based on the skeletal markers employed. Instead, it is likely that cultural factors were more often used to establish who was at risk of becoming a vampire and who was not. For example, newcomers to the community may have been viewed as outsiders and were thus seen as being at greater risk for vampirism upon their deaths [4].

Additionally, a recent pilot study examined cranial morphometrics of 11 individuals from the Drawsko 1 site, including two of the deviant burials (60/2010 and 6/2012) (34). While results indicated a moderate amount of cranial variation overall, potentially reflective of genetic admixture, one of the deviant burials (6/2012) was classified outside the range of normal variation within the Drawsko sample [34]. These results suggest that this individual may have been afforded different mortuary treatment as a result of her “outsider” status. However, this lack of consistency between the two individuals assessed suggests that the catalyst(s) prompting a vampire burial may be the result of a variety of social and cultural influences.

Apotropaic rites involving vampires have been reported not only at Drawsko but across eastern Europe during both the medieval and post-medieval periods (**Table 1**) [35]–[43]. However, while reports of such deviant burials have dominated headlines, few publications formally describing these finds exist [5],[6],[17],[27],[39]. Nevertheless, this preliminary assessment illustrates similarities in interment strategies and apotropaic inclusions, the most common of which included decapitation, iron stake(s) driven through the body, and stones placed directly atop the body, all of which were meant to prevent the individual suspected of becoming a vampire from leaving its tomb and preying upon the living community.

**Table 1:** Deviant burials associated with vampirism across Eastern Europe as assessed through popular media reports and published literature, in chronological order.

Site	Country	Date(century AD)	Period	# of DeviantBurials	Description								Source
					Decapitation/Dismemberment	Stake/Nailsin body	Stone in/beneathjaw	Stoneson body	Bound limbs	Sickle acrossbody	Burningevent	Coffin w/ironbars	
Celakovice	Czech Republic	10–11th	Medieval	14	X	X		X					[35]
Cedynia 2a	Poland	10–12th	Medieval	24+	X			X	X		X		[5]
Dębczyn	Poland	10–13th	Medieval	1	X			X					[5]
Cedynia 2a	Poland	12th	Medieval	6	X	X		X					[5]
Perperikon	Bulgaria	13–14th	Medieval	1		X							[36]
Sozopol	Bulgaria	14th	Medieval	1		X							[37]
Debelt	Bulgaria	Not specified	Medieval	6		X							[37]
Veliko Tarnovo	Bulgaria	Not specified	Medieval	1					X				[38]
Lazzaretto Nuovo	Italy	16th	Post-Medieval	1			X						[39]
Gliwice*	Poland	16th	Post-Medieval	4	X								[40]
Kamien Pomorski	Poland	16th	Post-Medieval	1	X	X	X						[41]
Prostejov	Slovakia	16th	Post-Medieval	1	X			X				X	[42]
**Drawsko**	**Poland**	**17th**	**Post-Medieval**	**6**			**X**			**X**			[27]
Lesbos	Greece	19th	Ottoman	1		X							[43]

*Others have interpreted these as executed criminals.

## Population Heterogeneity and Migration in Post-Medieval Poland

Drawsko is located within *Wielkopolska* (“Greater Poland”), a region fundamental to the establishment of Poland as an independent country in the 10^th^ century, particularly as the first Polish ruler, Mieszko I, resided there [10]. *Wielkopolska*, as well as Poland as a whole, was known for its religious and cultural heterogeneity as a result of various military conquests, religious tolerance offered by the state since the times of Casimir the Great (AD 1310–1370), and land acquisition and union with Lithuania (AD 1569) to form the ethnically diverse Polish-Lithuanian Commonwealth [44]. As a republic with an elected king whose power was limited and controlled by the Parliament, religious and political freedoms were an inherent part of the Commonwealth’s political system [44].

By the 17^th^ century, this diverse Polish population is estimated to have reached 7–8 million people [45],[46], in part a response to an influx of immigrants from across Europe. For instance, driven by economic and religious reasons, the immigration of Scots into mainland Europe began as early as the 14^th^ century and continued well into the 17^th^ century [47],[48],[49]. Tax records indicate that Scottish immigrants inhabited over one hundred localities within Poland but were largely centered in Warsaw, Poznań, Gdańsk, and Kraków [48],[49]. From the Balkan Peninsula to the south, the Vlach ethnic group began migrating northward during the medieval period and reached the mountainous region of southern Poland in the late 14^th^ century; assimilation with the local Ruthenian population gave rise to numerous, distinct ethnic groups still inhabiting the region today [50]. Furthermore, the extension of Polish territory to the east and its subsequent union with Lithuania (AD 1386) and the Grand Lithuanian Duchy (AD 1569) to form the Polish-Lithuanian Commonwealth contributed greatly to the multi-ethnic nature of this nation and resulted in the regional movement of Tatars, Armenians, Latvians, and Ruthenians into Poland [44].

From the west, German and Flemmish immigration into Poland started as early as the 13^th^ century, with the largest conglomeration of migrants settling in the Kraków area [45],[46]. At that time, Kraków – capital of the Polish-Lithuanian superpower, seat of the famous university, and a large center of commerce – was particularly enticing to German settlers, including wealthy patrons of the arts who successfully attracted German painters and sculptors to the region, as well as talented craftsmen and printers [51]. Also from the west, Mennonites from what is now the Netherlands and northern Germany (Friesland) were given religious freedom and privileges such as rent and tax concessions from the Polish state beginning around the 16^th^ century [44]. Well known for their abilities to drain marshlands for habitation and agriculture, migrants were initially brought to Poland to reclaim marshy areas on the alluvial delta of the Vistula River, and by the mid-17^th^ century, large migrant communities had settled around Włocławek, Świecie, Toruń, Malbork, and Chełm [44]. In addition, Jews expelled from their homelands or fleeing persecution flocked to Poland from western Europe in the aftermath of the Crusades throughout the 14^th^ and 15^th^ centuries [52].

Drawsko itself was located within the Wieleń Estate, a royal property sparsely populated until AD 1515, when a shift to private ownership instigated the rise of a number of new villages throughout the 16^th^ and 17^th^ centuries [53]. These villages – located in both *sołtysie* (forested areas) and *olęderskie* (marshlands) – were comprised not only of Pomeranian locals but also by settlers from the west, including Brandenburg and the Netherlands, seeking improved socioeconomic conditions and religious freedom [53]. While the most dramatic colonization of the Wieleń area began in the 1580s when the estate was owned by the Górka family, another wave of immigration took place in the early 18^th^ century in response to the devastating cholera epidemics throughout *Wielkopolska* and other lands of the Crown [53]. Nevertheless, a brief respite from colonization occurred between AD 1750–1760 – corresponding to the beginning of the demise of the feudal system – during which time many local peasants were relocated from Wielkopolska to Brandenburg [53].

The overwhelming historical evidence for migration to Poland – as well as the diverse composition of its population by the post-medieval period – illustrates that the inhabitants of Drawsko would likely have dealt with issues of immigration and witnessed the growth of an increasingly multi-ethnic community during the 17^th^ century. However, how this rural village may have adapted or negotiated its changing social identity is less clear, particularly with regards to the treatment (in life and in death) of outsiders entering the area. Because outsiders were considered to be more susceptible to vampirism after death [4], it was hypothesized that those targeted for apotropaic burial rites were migrants to the Drawsko area. While differences in ethnic identity could not be readily discerned from the Drawsko 1 interments, non-local migrants to a region may be identified using radiogenic strontium isotopes incorporated into human skeletal tissues.

## Strontium Isotopes and Geologic Variability

Strontium isotope analysis (^87^Sr/^86^Sr) of archaeological human dental enamel enables us to investigate patterns of residential mobility for those interred at the Drawsko 1 cemetery, and in particular, permits an examination of the geographic origins of those individuals suspected of vampirism and selected for apotropaic burial rites. While the abundance of stable ^86^Sr isotopes remains constant through time in the geologic environment, radiogenic ^87^Sr isotopes result from the natural decay of ^87^Rb (rubidium) in igneous rock and thus increase in number over time [54],[55]. Subsequently, older rocks such as granite possess more ^87^Sr isotopes and elevated ^87^Sr/^86^Sr values relative to younger volcanic rocks like basalt [56]. In addition to age, the initial amount of Rb/Sr found in a particular mineral type also plays a role in resultant ^87^Sr/^86^Sr ratios [57].

Strontium isotopes enter into local ecosystems through the weathering of bedrock, disseminated from soils and groundwater into flora and fauna [58]. Because strontium is structurally similar to calcium, as humans consume these plants and animals, small amounts of strontium absorbed by the intestines substitute for calcium in the formation of enamel and bone hydroxyapatite [59]. Strontium uptake into the human skeleton is primarily determined by these consumed foods, and because the ^87^Sr/^86^Sr ratios within these products are a direct reflection of the distinct isotopic composition of a particular region’s underlying geology, biogeochemical signatures in human dental enamel (which form only during childhood) offer a useful means of evaluating childhood geographic residence and mobility in the past [56]. However, factors including geologic heterogeneity (due to variable mineral ages and types in a given area) and differential weathering of bedrock make measuring bioavailable strontium using soil samples alone problematic; instead, animal values more broadly reflect available strontium in that environment and more closely approximate local ^87^Sr/^86^Sr ratios accessible to humans [56]. In this way, faunal ^87^Sr/^86^Sr values can be used to define locally bioavailable strontium for humans inhabiting a specific region – by taking the mean ± 2 standard deviations (s.d.) – and to detect non-local individuals whose strontium signatures fall outside of these designated limits of locality [56],[59].

Poland can be roughly divided geologically into northern and southern halves. The northern portion of Poland is dominated by the North European Plain, which sits between the Central European Highlands to the south and the Baltic Sea in the north. These lowlands, also encompassing Belgium, Denmark, the Netherlands, and northern Germany, are comprised of Cenozoic sediments deposited in the Quaternary period, including coversands, loess, and glacial gravel [60]. Conversely, the southern uplands are primarily represented by the Carpathian Mountains in the south and the Sudetes in the southwest. These formations display considerable geologic heterogeneity, containing a combination of volcanic basalts, limestone, and granites. From the Sudeten range, Blusztajn and Hart [61] report Tertiary basalts with depleted ^87^Sr/^86^Sr ratios (0.7032–0.7037) relative to older rocks in the region, including Triassic and Cretaceous limestones (0.707–0.708) [62] as well as old granites expected to produce even higher strontium isotope signatures [60].

Soil samples from across Poland produce a mean ^87^Sr/^86^Sr ratio of 0.709 and range from 0.7069–0.7123, overlapping with values from the adjacent German plain (0.7086–0.7109) but exhibiting some elevated ratios (0.711–0.713) not seen in the west [60],[63],[64]. Still higher values (0.71427) are recorded at Szczepankowice in southwestern Poland [65]. Surface water (0.7078–0.7096) throughout Poland and from the Vistula River (0.7095) also fall within these ranges, although these, along with ^87^Sr/^86^Sr values from wheat (0.7090–0.7106) and butter (0.7088), do not approach the higher ratios recorded in soil extracts from both the north and the south [63,66,67]. Recent strontium isotope analyses of faunal and human samples from Poland [60,65,68] are discussed further below (see Discussion).

## Materials and Methods

Bone from multiple species of modern local fauna (n = 6) was sampled to estimate ^87^Sr/^86^Sr bioavailability at Drawsko, with local values reflective of the range produced by two standard deviations on either side of the mean [56]. Animals sampled as part of this study included hare (n = 3), mice (n = 2), and fox (n = 1). Archaeological fauna were not present within the post-medieval mortuary context and were thus unavailable for sampling.

Enamel from a total of 60 human individuals (of the 285 burials excavated from 2008–2012, or 21% of excavated graves) dating to the post-medieval period were sampled from the cemetery at Drawsko, including 29 adult females, 29 adult males, and 2 immature individuals, or subadults. The two subadults (29/2008 and 6/2012) were included because they represent two of the six vampire burials recovered from the cemetery; the remaining four adults include three females and a single male. Dental enamel from a single permanent molar was analyzed from each individual, and depending on availability and tooth preservation, included the first molar (M1 mineralization occurs *in* *utero* until approx. 4.5 years of age), second molar (M2 – approx. 2.5–8.5 years of age), or third molar (M3 – approx. 8.5–15.5 years of age) [69]. An edentulous older female individual (60/2012) representing the fifth (of six total) vampire burial unearthed at Drawsko could not be analyzed due to antemortem tooth loss. Of the 60 individuals sampled here, 22 coins were distributed between females (n = 14), males (n = 7), and a subadult (n = 1). Three vampire burials (6/2012, 24/2009, and 49/2012) of six contained a coin recovered beside the left side of the head, while a fourth (60/2010) was found with copper staining inside the mouth, but no coin.

The excellent preservation of these remains permitted estimates of both age and sex for almost all sampled individuals. Sex estimation techniques included observations of features on both the os coxae and crania [70], but were not attempted for the younger of the two subadults included in this study. Subadult age was estimated using dental development as well as the timing of dental eruption and epiphyseal fusion [71]. Adult age estimates were based on observations of age-related changes to the pubic symphysis and auricular surface [70]. Human skeletal remains are currently housed at the Town Hall in the modern village of Drawsko, Poland.

Surface enamel from each tooth was mechanically removed to eliminate diagenetic contamination before extracting 3–5 mg of enamel powder for analysis, obtained using a Dremel Tool with an attached carbide drill bit. Sample preparation followed protocol outlined by Perry et al. [72]. Preparation and analysis of all samples took place at the University of North Carolina at Chapel Hill Isotope Geochemistry Laboratory. Enamel samples dissolved in 3.5 M HNO_3_ underwent column extraction using EiChrom Sr-Spec resin, with the resultant extract treated with 0.1 M H_3_PO_4_. Liquid samples were dried, re-dissolved in TaCl_5_, and placed onto rhenium filaments where they were dried down with an electrical current. Radiogenic strontium isotope ratios were analyzed on a VG Micromass Sector 54 thermal ionization mass spectrometer (TIMS) in quintuple-collector dynamic mode. An internal ratio of ^86^Sr/^88^Sr = 0.1194 was used to correct for mass fractionation, and for the NBS-987 standard, ratios are reported relative to a value of 0.710270±0.000014 (2σ). Internal precision for strontium runs is typically 0.000012–0.000018% (2σ) standard error based on 100 dynamic cycles of data collection.

Due to unequal sample sizes for deviant/non-deviant burials, as well as non-normal data distributions, non-parametric statistical tests (including Mann-Whitney U and Kruskal Wallis) were conducted using SAS 9.3. Specimen numbers are comprised of burial number and year of excavation and are listed in **Table 2**. The Drawsko I skeletal collection is stored in Poland at the District Office of Drawsko (URZĄD GMINY DRAWSKO, ul. Powstańców Wlkp. 121, 64–733 Drawsko). No permits were required to transport or analyze the Drawsko teeth as part of the current study, although an official permission letter was provided by the Slavia Project and International Project Coordinator Dr. Marek Polcyn. The excavation permit (Permit No. 42/2014/c) for the site of Drawsko I was issued by the Regional Conservator Office in Piła, Poland.

**Table 2:** Strontium isotope ratios for post-medieval human enamel samples from Drawsko, Poland.

Burial #	Year Excavated	Age Category	Age (Years)	Sex	Tooth	^87^Sr/^86^Sr
7	2010	Adult	40–49	M	RM^1^	0.70999
5	2011	Adult	27–32	F	RM_2_	0.71002
1	2012	Adult	30–50	M	RM_1_	0.71006
46	2010	Adult	30–34	F	LM_1_	0.71011
64	2011	Adult	35–39	M	RM_3_	0.71013
4	2011	Adult	20–30	M	LM^1^	0.71026
12	2010	Adult	30–34	F	RM^1^	0.71034
30	2011	Adult	40–44	F	LM^2^	0.71045
26	2011	Adult	40–49	M	RM^1^	0.71046
35/40A - Box 1	2009	Adult	25–29	M	LM_1_	0.71065
**49**	**2012**	**Adult**	**30–39**	**F**	**LM^1^**	**0.71072**
**29**	**2008**	**Subadult**	**12–15**	**I**	**LM^1^**	**0.71077**
27	2008	Adult	30–39	M	LM_1_	0.71077
26	2008	Adult	40–49	M	RM_1_	0.71078
28	2009	Adult	35–44	F	RM^1^	0.71080
24	2012	Adult	18–30	F	RM_1_	0.71084
10	2012	Adult	30–50	F	RM^1^	0.71085
31	2009	Adult	30–39	M	RM_1_	0.71090
40	2011	Adult	40–44	F	RM^2^	0.71096
37	2012	Adult	30–50	M	RM^1^	0.71099
8	2011	Adult	32–37	F	RM^1^	0.71102
2	2012	Adult	50+	F	LM^1^	0.71103
58	2011	Adult	25–29	M	LM^1^	0.71103
22	2008	Adult	50–59	F	RM_1_	0.71104
25	2009	Adult	50–59	F	RM^1^	0.71104
2	2011	Adult	30–34	M	LM^2^	0.71109
21	2011	Adult	28–34	F	LM^1^	0.71111
5	2012	Adult	30–50	F	RM^3^	0.71115
23	2009	Adult	40–44	F	RM^1^	0.71116
14	2012	Adult	30–50	F	RM_1_	0.71117
20	2009	Adult	25–29	F	LM^1^	0.71118
66	2010	Adult	30–34	F	LM^1^	0.71119
1	2011	Adult	25–29	F	RM_2_	0.71123
19	2008	Adult	40–44	M	RM_1_	0.71127
45	2008	Adult	50–59	F	LM_3_	0.71127
35	2008	Adult	40–44	F	RM_1_	0.71128
11	2011	Adult	17–23	M	LM^1^	0.71128
**28**	**2008**	**Adult**	**35–44**	**M**	**LM_2_**	**0.71131**
17	2009	Adult	25–29	F	LM^1^	0.71131
25	2011	Adult	25–29	M	LM^1^	0.71132
32	2011	Adult	27–35	M	RM^1^	0.71136
13	2012	Adult	30–50	F	LM_1_	0.71136
12	2011	Adult	40–44	M	LM_3_	0.71139
**6**	**2012**	**Subadult**	**14–19**	**F**	**RM_1_**	**0.71139**
32	2012	Adult	18–30	F	LM_2_	0.71139
63	2010	Adult	35–39	M	LM_3_	0.71142
31	2012	Adult	18–30	M	RM^1^	0.71146
7	2009	Adult	25–35	M	RM_1_	0.71146
23	2012	Adult	30–50	M	LM^1^	0.71148
**24**	**2009**	**Adult**	**35–39**	**F**	**RM^1^**	**0.71156**
36	2012	Adult	30–50	M	RM_2_	0.71157
57	2011	Adult	30–34	F	RM_1_	0.71169
42	2012	Adult	30–50	M	RM_2_	0.71176
35	2012	Adult	18–30	F	RM_1_	0.71178
70	2010	Adult	40–44	M	RM^1^	0.71178
66	2011	Adult	35–39	M	LM_1_	0.71183
49	2010	Adult	40–44	F	RM_1_	0.71199
40	2008	Adult	35–44	M	RM_1_	0.71230*
36	2011	Adult	30–34	M	LM^1^	0.71283*
82	2010	Adult	25–29	M	RM_1_	0.71301*

Vampire burials are represented in bold text, while outliers are denoted with an asterisk.

## Results

**Figure 3:**
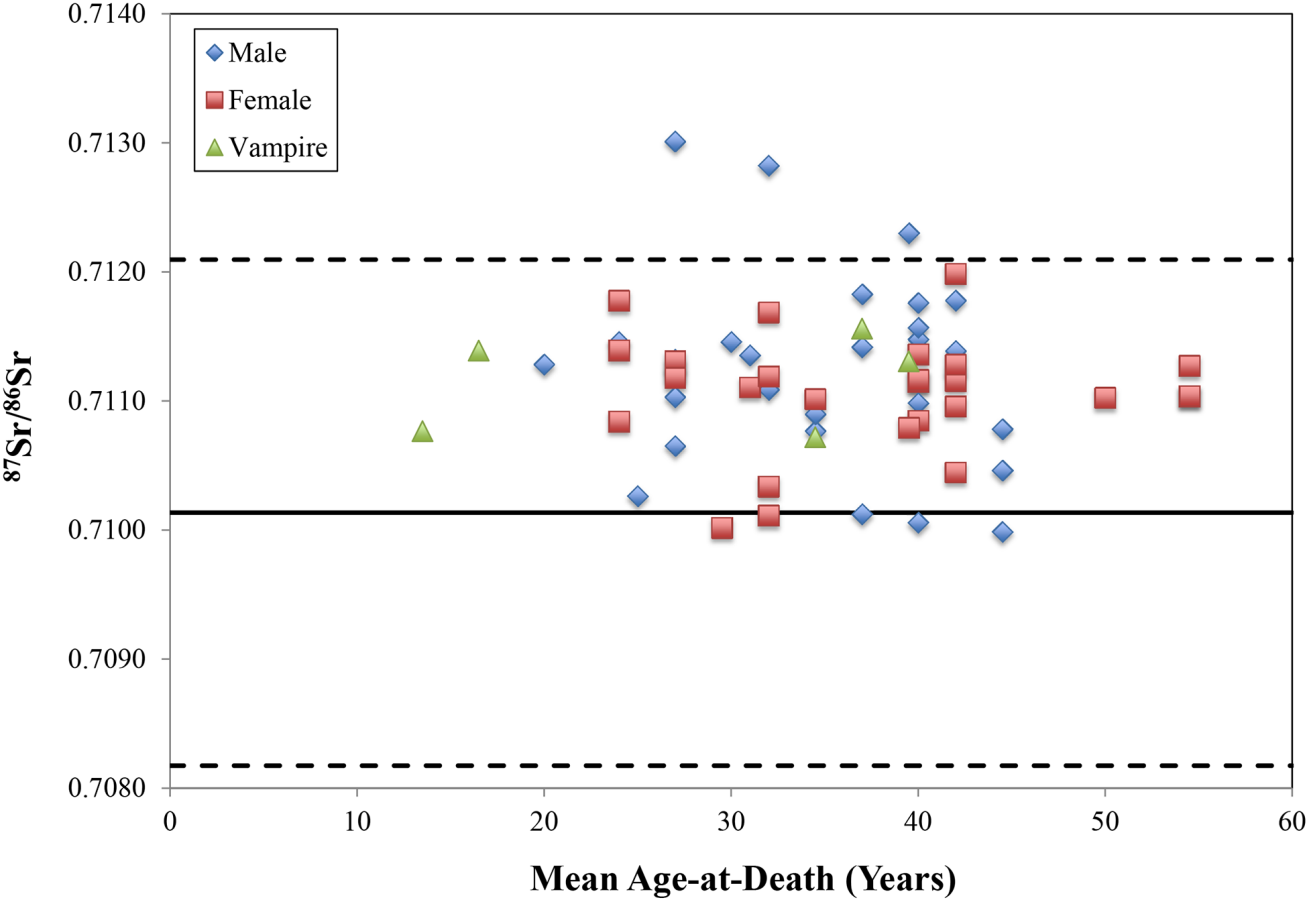
Strontium isotope ratios from human dental enamel at Drawsko. Age midpoints were taken from estimated age ranges for each individual. The area between the dotted lines represents expected bioavailable strontium for the site based on modern faunal ^87^Sr/^86^Sr values, while the solid line represents the mean faunal ^87^Sr/^86^Sr ratio.

Fauna (n = 6) from Drawsko exhibit a mean ^87^Sr/^86^Sr value of 0.7101±0.0012 (2σ), which was used to generate a local baseline ranging from 0.7082 to 0.7121 (**Table 3** **,** **Figure 3**). By species, hare (n = 3) display an average ^87^Sr/^86^Sr ratio of 0.7095±0.0005 (1σ), while mice (n = 2) show a more elevated mean of 0.71131±0.00002 (1σ). A lone fox sample (^87^Sr/^86^Sr = 0.7097) produced a value between these two.

**Table 3:** Strontium isotope ratios for modern local fauna from Drawsko, Poland.

Site	Period	Fauna	Bone	^87^Sr/^86^Sr
Drawsko	Modern	mouse	mandible	0.711296
Drawsko	Modern	fox	tibia	0.709740
Drawsko	Modern	mouse	os coxa	0.711328
Drawsko	Modern	hare	frontal	0.709752
Drawsko	Modern	hare	femur	0.709840
Drawsko	Modern	hare	femur	0.708853

Human dental enamel (n = 60) demonstrates an overall, average ratio of 0.7112±0.0006 (1σ) (**Table 2**, **Figure 3**). For adults (n = 58; 0.7112±0.0006, 1σ), female ^87^Sr/^86^Sr values (n = 29; 0.7111±0.0004, 1σ) do not differ statistically from males (n = 29; 0.7112±0.0007, 1σ) (p = 0.26; Mann-Whitney U). Subadults (n = 2; 0.711±0.004, 1σ) share a similar mean, but small sample size prevents statistical comparison with adults. Individuals suspected of vampirism that underwent apotropaic burial rites (n = 5) produce a mean ^87^Sr/^86^Sr ratio of 0.7112±0.0004 (1σ) and were not significantly different from either males or females interred within non-deviant mortuary contexts (p = 0.54; Kruskal Wallis).

Three individuals (40/2008; 82/2010; 36/2011) fell outside local strontium isotope ratios for Drawsko, ranging in ^87^Sr/^86^Sr value from 0.702301 to 0.713013 (**Figures 3** **&** **4**). All were adult males with age-at-death estimated at between 25–44 years, and two (40/2008; 36/2011) of these three were interred with coins.

**Figure 4:**
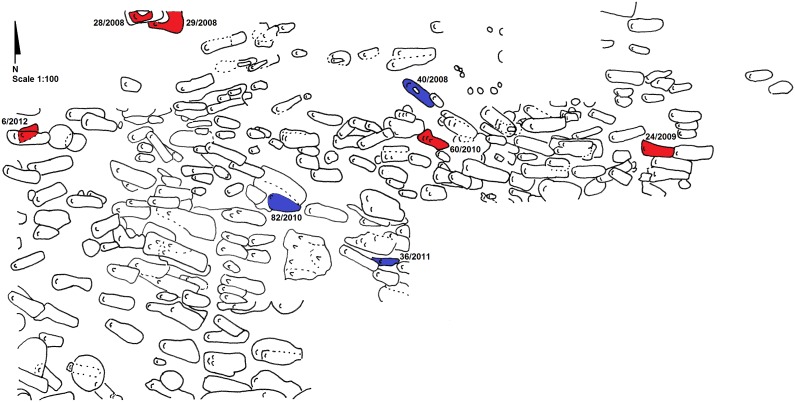
Map of the Drawsko 1 cemetery (Scale 1∶100). Burials highlighted in red represent vampire graves, while those highlighted blue represent outliers as identified by strontium isotope analysis.

## Discussion

### Strontium Isotopes at Drawsko

The majority (n = 57) of individuals analyzed from the Drawsko 1 cemetery exhibit strontium values consistent with local ranges as defined by modern fauna, suggesting that they likely resided in/around Drawsko during childhood, when enamel mineralization takes place. Such an overall lack of mobility points to the establishment of settlements in the 16^th^–17^th^ centuries throughout the Wieleń Estate (where Drawsko was located) *before* the cemetery was in use, when non-local migrants would have joined local Pomeranian communities to the west in populating the area. Moreover, of the five sampled individuals interred as vampires with sickles across their bodies and/or rocks beneath their jaws, all displayed local strontium isotope ratios, causing us to reject the hypothesis that these individuals were intentionally buried with apotropaics meant to prevent them from returning as the undead because of their ‘outsider’ status as non-local migrants to the area.

**Figure 5:**
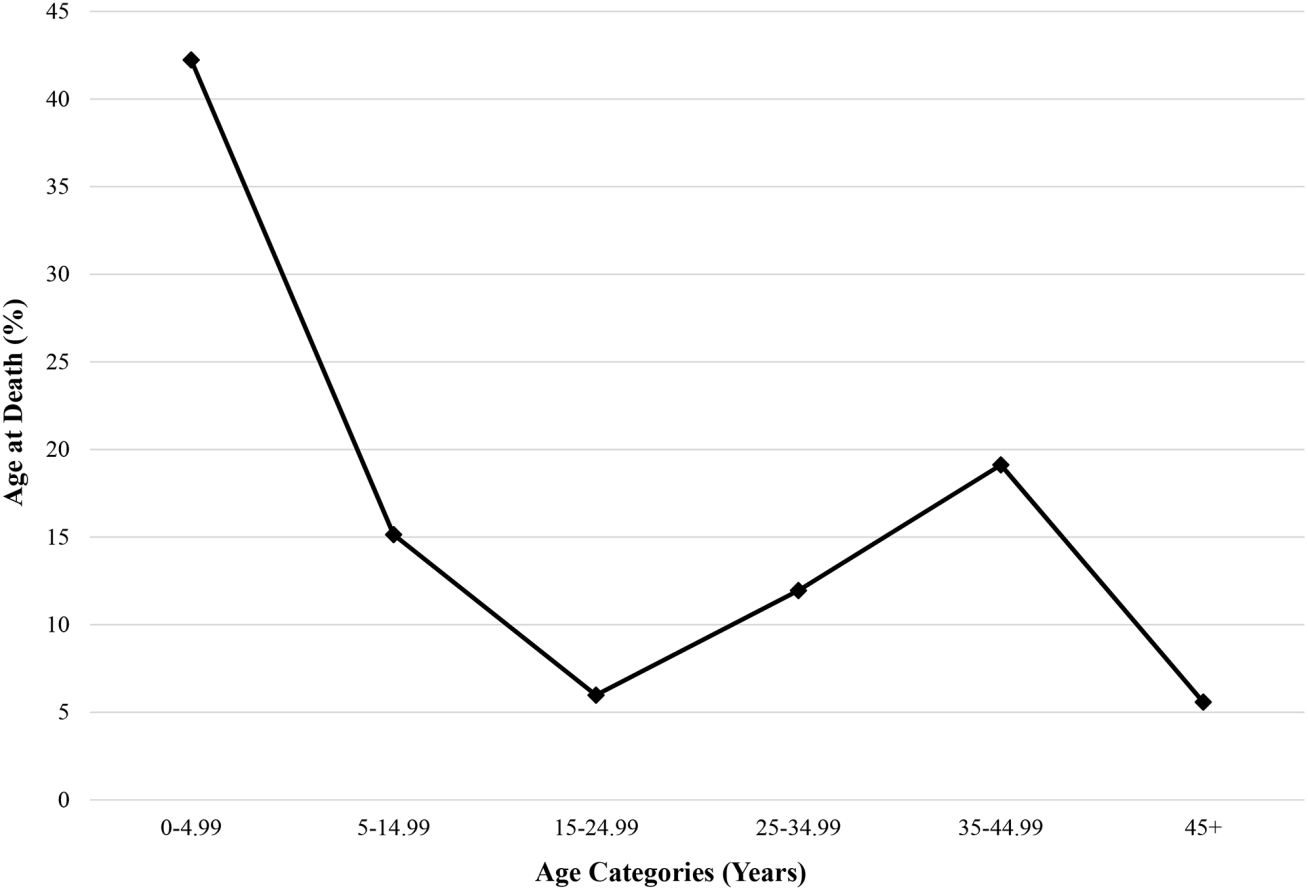
Demographic profile for the Drawsko 1 site.

Why, then, were these local individuals targeted when others were not? In addition to being an outsider, a multitude of attributes associated with vampirism also made *locals* susceptible to accusation, possibly resulting in apotropaic burial at Drawsko. Individuals ostracized during life for their strange physical features, those born out of wedlock or who remained unbaptized, and anyone whose death was unusual in some way – untimely, violent, the result of suicide, or even as the first to die in an infectious disease outbreak – all were considered vulnerable to reanimation after death [4],[5],[13],[16],[18]. In particular, historic records describe multiple cholera epidemics that swept through Poland throughout the 17^th^ century as a result of contaminated water [28]. While cholera kills too quickly to produce pathognomonic changes visible on the skeleton [73], the unusual characteristics of the Drawsko 1 cemetery – including the absence of a church (from excavations to date), the seemingly random placement of multiple overlapping graves (see **Figure 4**), and poorly-fitted coffins (perhaps from rushed interment) – have hinted that the site may represent an epidemic burial ground resulting from a cholera outbreak [27]. Nevertheless, the enormity of the cemetery serving a small village like Drawsko calls this claim into question, making it more probable that while some of the interments at Drawsko 1 may indeed represent individuals killed by infectious disease, most do not [27]. Moreover, an examination of the mortality profile for 251 individuals from the site whose ages could be reliably estimated reflect an attritional cemetery, rather than a catastrophic cemetery (**Figure 5**), in which mortality peaks in the youngest ages (<5 years of age) followed by a secondary peak in older adults [74],[75]. Despite this, the possibility remains that the six individuals who underwent anti-vampiristic mortuary rites at Drawsko were the first to succumb to the disease during a series of outbreaks, instigating apotropaic action by the living. However, given the small settlement size, a separate epidemic cemetery was likely unnecessary, and individuals who may have died from cholera were simply interred in the communal cemetery.

While none of the five interments that included these apotropaics were identified as immigrants to the region, the presence of three males whose ^87^Sr/^86^Sr ratios exceed the upper limit of locally bioavailable strontium demonstrates that some residential mobility was indeed taking place during the time in which the cemetery was utilized by the community at Drawsko. Interestingly, these individuals were interred with no indication that their social or ethnic identity differed from that of the larger, local community – no foreign grave goods were recovered from their graves, nor were other, differentiating mortuary treatments apparent. This may suggest that – while identity is undoubtedly a multi-faceted social construct [e.g, see 76] – the role that migration may have played in the lives of these three individuals was not significant enough to be represented in death and/or was masked by other factors, including burial rites specific to Catholicism.

The identification of only males as non-local migrants to Drawsko may also shed light on what segments of society were afforded the ability to move from one place to another, particularly as all females examined in the group exhibited local values. While textual records do not specifically describe whether single individuals or entire families migrated and settled in Poland during this period, the biogeochemical results from Drawsko indicate that men were more mobile. The timing of this period of mobility is likewise unclear – while all three males were identified as non-local as a result of ^87^Sr/^86^Sr ratios from first molar enamel crowns that form up to 4.5 years of age, age-at-death ranged from 25–44. Future studies will examine individual life histories and the relationship between strontium isotope ratios in both enamel and ribs to determine when migration may have occurred into adolescence and/or adulthood.

### Interregional Distribution of Strontium Isotopes

**Figure 6:**
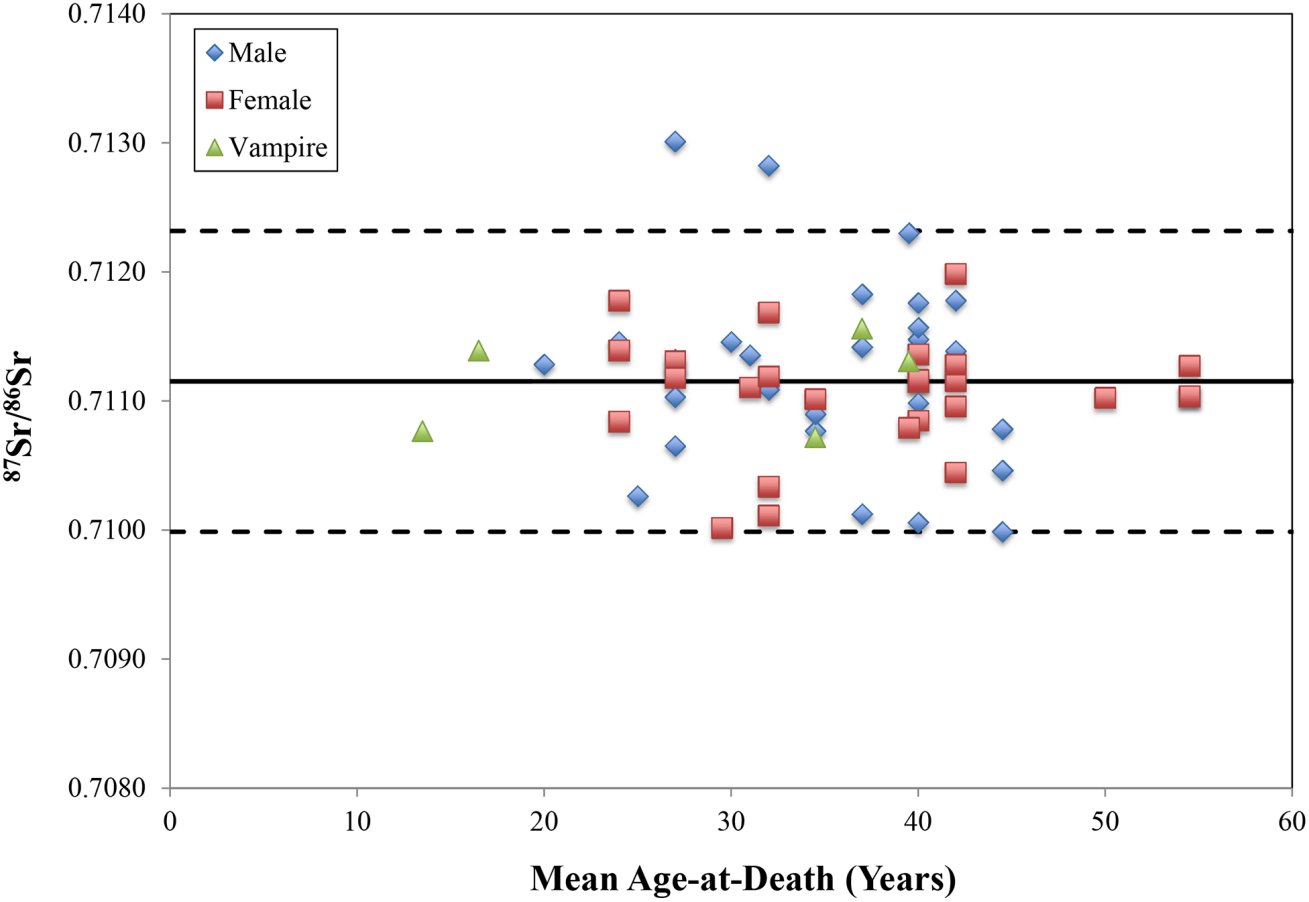
Strontium isotope ratios from human dental enamel at Drawsko, with a local baseline now derived from human, and not faunal, ^87^Sr/^86^Sr values.

Despite a wide breadth of local values (0.7082–0.7121) produced by multiple species of modern fauna, human ^87^Sr/^86^Sr ratios at Drawsko were largely limited to the upper stratum of this range, with all but five individuals falling above the mean faunal value of 0.7101 (**Figure 3**). This suggests that strontium input for humans at Drawsko differed somewhat from that biologically available to the small fauna analyzed here (a difference also echoed between the various species of fauna themselves, which produced a bimodal distribution of ^87^Sr/^86^Sr values making an assessment of locality using standard deviation difficult). Differences in diet or lifestyle provide the most likely explanation for this disparity; additionally, because archaeological fauna were not available for testing, modern fauna may have been exposed to sources of strontium not encountered in the post-medieval period, including fertilizers [59]. Subsequently, human ^87^Sr/^86^Sr values themselves may provide a more accurate local range for Drawsko, with a human average of 0.711±0.001 (2σ) producing ratios ranging from 0.7100 to 0.7123 (as with fauna, taking two standard deviations on either side of the mean) (**Figure 6**). Regardless of this shift in local ^87^Sr/^86^Sr values, the results are largely the same, with almost all individuals producing ratios local to the area except for two outliers – 82/2010 and 36/2011 – as opposed to three (with individual 40/2008) as identified by the local faunal baseline.

When examining local values produced by humans and/or fauna, a clearer picture emerges regarding the distribution of bioavailable radiogenic strontium isotopes throughout northern and central Europe. Significant overlap in local ^87^Sr/^86^Sr ratios occurs at sites located in Austria (0.7090–0.7100) [77], Germany (0.7082–0.7103) [64],[77]–[80], Denmark (0.7090–0.7108) [64], and Hungary (0.7091–0.7107) [77],[81], although the lowest values in the region derive from individuals sampled in Bavaria in southeastern Germany. While some overlap continues into the Czech Republic (0.7101–0.7109 [77] and Poland (0.7099–0.7147) [60,65,68], this region in general possesses ^87^Sr/^86^Sr values elevated (+0.7100) relative to western and central Europe. Nevertheless, such overlap indicates that more non-locals may in fact be present at Drawsko than can be identified by ^87^Sr/^86^Sr values alone. Moreover, it must be emphasized that because these local values were derived from particular sites, they do not necessarily represent the geologic heterogeneity present throughout Europe, and are likely not representative of the total variation present in each country as a whole.

Considerable isotopic heterogeneity identified throughout Poland likely reflects geologic variability contrasted between the northern plains and the southern uplands. As reported by Buko and colleagues [60], archaeological human dental enamel ^87^Sr/^86^Sr ratios from across the northwest (0.7099–0.7113), where Drawsko is located, largely fall within local ranges for Drawsko, although three presumed outliers from Dębczyno (n = 2; 0.7148–0.7151) and Pyrzyce (n = 1; 0.7136) may point to additional examples of residential mobility throughout the region. These values from the northwest contrast sharply with local faunal ratios from central (0.7120–0.7135) [60] and southwestern (0.7147) [65] Poland, likely a result of the exposed older bedrock of the southern uplands, including the Carpathanian and Sudeten ranges. Additional Early Bronze Age human ^87^Sr/^86^Sr values (0.7125–0.7135) interpreted as local to Silesia in southwestern Poland by Pokutta [68] exhibit similarly elevated ranges relative to the north.

While additional strontium isotope data from across Europe is needed, it appears possible that the three non-local immigrants present at Drawsko (^87^Sr/^86^Sr = 0.7123–0.7130) may have originated from central or southwestern Poland.

## Conclusions

The geographic origins of those interred in the post-medieval cemetery site of Drawsko 1 were investigated in order to assess whether apotropaic practices were specifically applied to non-local migrants thought to have the potential to become vampires due to their outsider status. Historic records describe characteristics that make one more susceptible to vampirism in death; it was these qualities that the living evaluated to determine the apotropaic inclusions necessary to accompany the deceased, as a means to prevent them from rising from their tombs and feeding on the living. While many of these characteristics (e.g., being unbaptized, practicing witchcraft) represent aspects of social identity and judgment difficult to reconstruct today using only human remains, other facets of identity are embedded into the skeleton itself, including the incorporation of stable isotopes into human bone and enamel during life. Specifically, the analysis of radiogenic strontium isotopes within dental enamel permit an examination of residential mobility and immigration – these biological markers can help explicate social definitions of the ‘outsider’ and determine whether individuals targeted for apotropaic rites were in fact non-locals.

The majority of individuals sampled exhibited ^87^Sr/^86^Sr values consistent with local ranges of bioavailable strontium for the area, suggesting that most of those interred in the cemetery had in life resided in or around Drawsko since early childhood. While geologic homogeneity across much of the North European Plain may mask some migrants to the region who were subsequently buried in the Drawsko 1 cemetery, three adult males did show elevated ^87^Sr/^86^Sr ratios that fell outside the upper limit of this local range, demonstrating that migration to Drawsko was still taking place. Extensive isotopic mapping of north and central Europe by previous bioarchaeological studies has identified central Poland as a potential candidate for the origin of these individuals, although more work in Poland and Eastern Europe is required to more fully comprehend the complexity of strontium isoscapes and bioavailability across this region.

The ^87^Sr/^86^Sr values of those selected for deviant burial (defined as such by the presence of sickles lain across the neck or abdomen and stones placed beneath the mandible) are also indicative of local origins. Subsequently, these individuals were not suspected of becoming vampires due to their identity as non-locals, but instead, were distrusted within some other, additional societal context as members of the local community. Evidence of widespread cholera epidemics throughout the region during the post-medieval period may offer an alternate explanation as to the reason behind these apotropaic mortuary practices, as – in addition to being an outsider – the first person to die from an infectious disease outbreak was presumed more likely to return from the dead as a vampire. However, because cholera kills quickly and does not leave behind visible markers on the skeleton, it is unclear if this is the case at Drawsko.

Strontium isotope data from the individuals interred at the cemetery at Drawsko provide new bioarchaeological perspectives into deviant burials and the motivations behind these unusual inhumation techniques. Moreover, this biogeochemical analysis highlights the complexities of examining social identity in the past, and how the construction of identity may be intertwined with biological processes contributing to the makeup of skeletal tissues. Future work will include stable oxygen and carbon isotope analysis of enamel and bone samples (as well as strontium isotope analyses of bone) to further investigate residential mobility, geographic origins, and dietary variability between normative and deviant burials throughout the life history of those inhabiting northwest Poland during the post-medieval period.
